# A longitudinal study of the distance that young people walk to school

**DOI:** 10.1016/j.healthplace.2014.10.013

**Published:** 2015-01

**Authors:** P. Chillón, J. Panter, K. Corder, A.P. Jones, E.M.F. Van Sluijs

**Affiliations:** a PROFITH “PROmoting FITness and Health through physical activity” research group, Department of Physical Education and Sport, Faculty of Sport Sciences, University of Granada, Ctra de Alfacar s/n, Granada 18071, Spain; b Medical Research Council Epidemiology Unit & UKCRC Centre for Diet and Activity Research (CEDAR), Institute of Public Health, University of Cambridge, Box 285, Addrenbooke׳s Hospital, Hills road, Cambridge CB2 0QQ, United Kingdom; c UKCRC Centre for Diet and Activity Research (CEDAR) & School of Environmental Sciences, Norwich Medical School, University of East Anglia, Norwich NR4 7TJ, United Kingdom

**Keywords:** Walking to school, Distance, Children, Adolescence

## Abstract

Walking or cycling to school has been associated with important health benefits. Distance between home and school is the main correlate of active commuting to school, but how far children walk to school and how this changes as children age is unknown. Mode of commuting and objectively-assessed distance to school were measured at 3 time points: aged 9/10 years, 10/11 years and 13/14 years. Data were analysed using ROC-curve analyses. With age, children walked further to school; the threshold distance that best discriminated walkers from passive commuters was 1421 m in 10-year-olds, 1627 m in 11-year-olds and 3046 m in 14-year-olds. Future interventions should consider the distance that young people actually walk.

## Highlights

• The distance walked to school changes when children age.
• The threshold distance that best discriminates walkers and passive commuters is 1.4 km for 10 years children and 3 km for 14 years adolescents.
• These threshold distances will help inform policy, practice and future intervention efforts.

## 1 Introduction

Active commuting to school provides an opportunity for increasing levels of physical activity on school days (Larouche et al., 2014). However, in many countries the prevalence of active commuting to school has declined in recent decades (Black et al., 2001; Buliung et al., 2009; McDonald, 2007; van der Ploeg et al., 2008). Understanding the correlates and determinants of active commuting to school aids the development of strategies to increase rates of active commuting in young people (Tudor-Locke et al., 2001). Travel distance has been shown to have the strongest association with active commuting to school, with shorter distances associated with higher rates of active travel (Panter et al., 2008; Pont et al., 2009), and is also associated with changes in active commuting (Hume et al., 2009; Panter et al., 2013). However, little evidence is available on the distance that children are willing to walk to school. In Belgian children, walkable distances of 1.5 km and 2 km for 11–12 year olds and 17–18 year olds, respectively, have been reported (D’Haese et al., 2011; Van Dyck et al., 2010), whereas an Irish study reported an acceptable walking distance of 2.4 km for 15–17 year olds (Nelson et al., 2008). However, how far younger children travel and how this changes when children grow older is unknown. Understanding the thresholds above which young people are less likely to walk to school may inform local and national governments in making policy decisions regarding supporting active commuting to school.

The aims of the current paper therefore are (a) to study the association between objectively-measured distance from home to school and mode of commuting from childhood to adolescence; and (b) to identify age-specific threshold distances below which young people are more likely to walk to school as opposed to using passive modes of transport.

## 2 Methods

### 2.1 Study design and setting

The SPEEDY study (Sport Physical Activity and Eating Behaviour: Environmental determinants in Young people) is a population-based longitudinal cohort study investigating factors associated with physical activity and dietary behaviour among children attending schools in the county of Norfolk, UK. The study design and procedures have been detailed elsewhere (Corder et al., 2014; Van Sluijs et al., 2008). Ethical approval was obtained from the University of East Anglia research ethics committee.

### 2.2 Participant recruitment

Participants were invited to participate on three separate occasions: in Year 5 (10 years, April–July 2007), Year 6 (11 years, April–July 2008), and Year 9 (14 years, April–August 2011). At age 10 years 2064 children participated, of which 2053 (99.4%) provided data on mode of commuting to school. At age 11 years, all 2064 original participants were invited to participate and 1019 (49.4% of the original sample) consented; 911 (44.1%) provided commuting data. At age 14 years, the 1964 baseline participants with valid home addresses at 11 years were invited; 480 (23.3% of the original sample) consented with 475 (23.0%) providing commuting data.

### 2.3 Measures

#### 2.3.1 Mode of commuting to school

Participants answered the same question at all measurements: “*How do you usually travel to school*?”, with four response categories: “(*a*) *by car*, (*b*) *by bus or train*, (*c*) *by bicycle*, *and* (*d*) *on foot*”. Use of car, bus or train was defined as “passive commuting” and cycling and walking were defined as “active commuting”. Most active commuters were walkers (i.e., there were 9.2%, 7.9%, 4.4% cyclists at each time point). Therefore, only walkers and passive travellers (car or public transport) were included in analyses.

#### 2.3.2 Distance from home to school

The objective measure of distance to school was estimated using a Geographic Information System Software package (ArcGIS 9.2, ESRI Inc). Parents provided home address details which were geo-referenced using Address Layer 2, a dataset that identifies precise locations for all registered addresses in Great Britain (Ordenance, Survey, 2006). If parents provided house names which did not exactly match the house names provided in Address Layer 2, the closest valid address was used. For those with missing street names, it was impossible to geolocate addresses. Travel distance was estimated for all participants by calculating the shortest route via the street network between each child׳s home and the nearest school entrance. As children attended the same school at 10 years and 11 years, distance was kept constant. Those moving house between 10 years and 11 years were excluded from the analyses at 11 years, as we were unable to verify their current school (*N*=42). Per age group, travel distance was categorised in percentiles and quintiles for the descriptive and binary logistic analysis respectively.

#### 2.3.3 Potential confounders

Age was calculated by date of birth at measurement dates; sex was self-reported at baseline. Height and weight were measured at 10 years and 14 years using standardized protocols. Body mass index (BMI) was calculated as kg/m^2^. The educational level of the main caregivers (hereafter “parents”) was self-reported using their age at leaving full-time education which was collapsed into 3 categories: <16 years, 16–18 years, and >18 years. The urban/rural status of the home was determined based on home location (Bibby and Shepherd, 2004).

### 2.4 Statistical analysis

Differences in baseline characteristics (BMI, sex, parental educational level and commuting mode) between participants with (*n*=911 and 475) and without (*n*=1153 and 1589) valid data at 11 years and 14 years, respectively, were tested using *t*-tests for normal continuous variables, non-parametric tests for non-normal continuous variables, and chi-squared tests for categorical variables. The association between travel mode to school and distance from home to school at each time point was studied using binary logistic regression. Travel mode was included as a binary dependent variable (walk vs. passive) and the distance from home to school (categorised in quintiles with longest distance as reference) as a categorical exposure variable, adjusting for sex, BMI, parent׳s educational level and urban/rural status.

The “threshold” distance for walking was calculated through the Receiver Operating Characteristic (ROC) curve analyses at all three time points. ROC curve analysis has been widely used in situations where the evaluation of discrimination performance is of great concern for the researchers (Komori and Eguchi, 2010). The area under the ROC curve is the most popular metric because it has a simple probabilistic interpretation and consists of two important rates: the true positive rate (or sensitivity) and the false positive rate (1-specificity). The larger the area under the curve (ranking from 0 to 1), the more discriminatory the test. Using the sensitivity and specificity obtained through the ROC-curves, the Youden index, which corresponds to the maximum vertical distance between the ROC curve and the diagonal line (Schisterman et al., 2005), was calculated. The Youden index corresponded to the distance (i.e. threshold distance) that best discriminates walkers from passive travellers. A sensitivity analysis was undertaken by repeating the analyses including only those who participated at all three time points (*n*=365).

## 3 Results

Drop-out analyses showed baseline differences between those in- and excluded. At age 11 years, those included had a lower baseline BMI (included vs. excluded: 17.9 vs. 18.4 kg/m^2^; *p*=0.004) and were more likely to be female (47.4% vs. 52.6%; *p*=0.002). At age 14 years, those included had parents with higher educational level (27.8% vs. 72.7%; *p*=0.006). No other differences were observed.

**Fig. 1:**
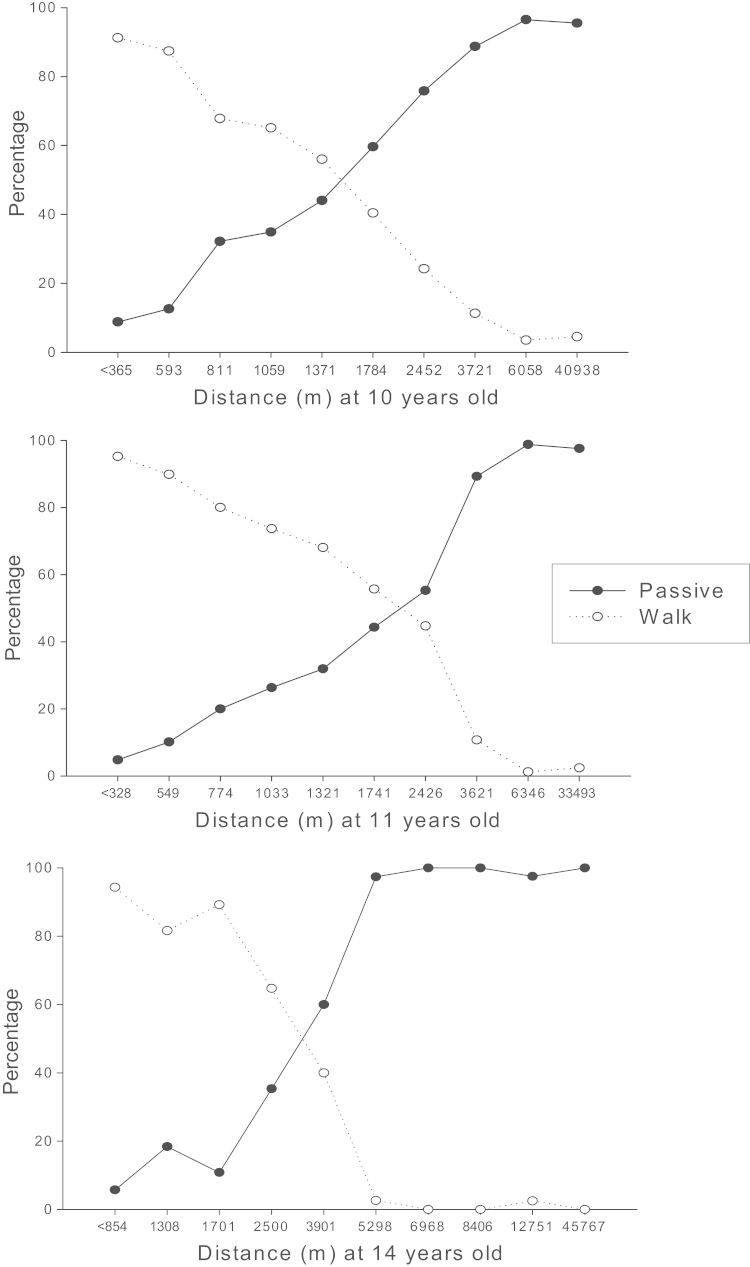
Percentage of walkers and passive commuters by distance from home to school (in percentiles) at 10 years (*n*=1826), at 11 years (distance home to school: all participants at 11 years except those 42 that moved from the measurement at 10 years; *n*=780) and at 14 years (*n*=372).

**Fig. 2:**
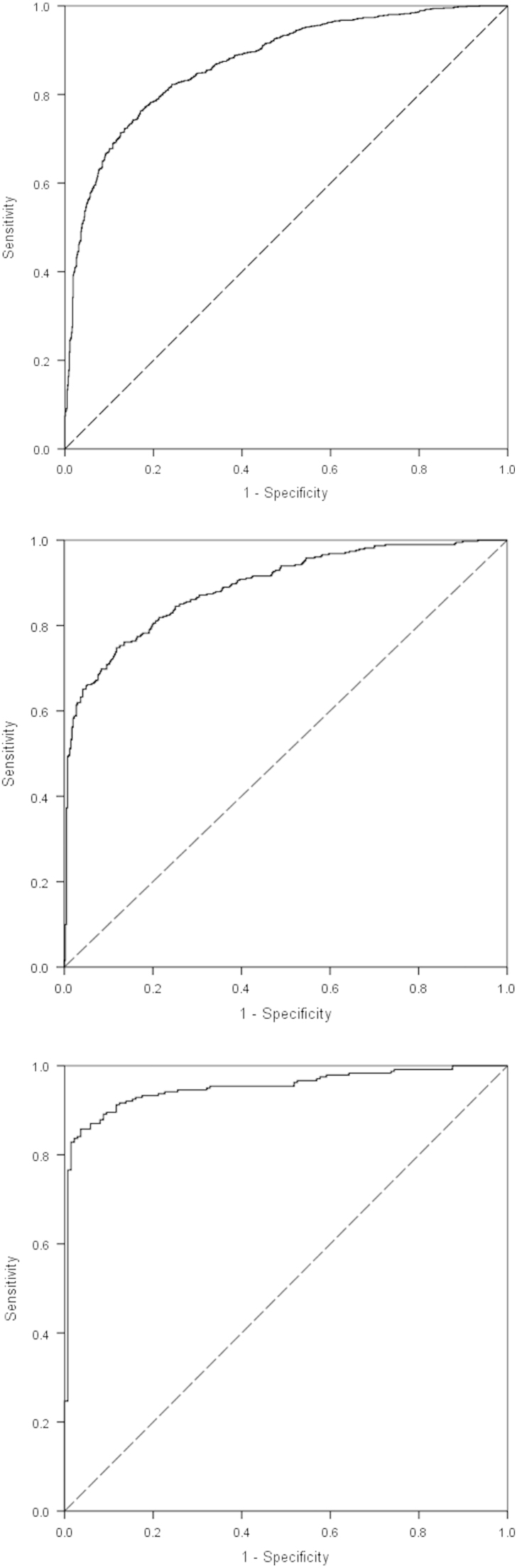
ROC curve analysis for walkers (negative) vs. passive (positive) commuters to school according to distance from home to school at 10 years (*N*=1825), at 11 years (*N*=788) and at 14 years (*N*=376). (a) ROC Curve at 10 years old (b) ROC Curve at 11 years old and (c) ROC Curve at 14 years old.

Table 1 presents the descriptive characteristics of the sample, whilst the percentage of walkers and passive commuters by distance are presented in Fig. 1. As expected, the percentage of children walking to school decreased with increasing distance. In adjusted logistic regression analyses, the association between travel distance and mode was statistically significant (*p*<0.001; Table 2). The age-specific ROC curves are shown in Fig. 2. The areas under the curve (standard error) were 0.872 (0.008), 0.891 (0.011) and 0.951 (0.011) (all *p*<0.001) at 10, 11, and 14 years respectively. The Youden indices points (sensitivity, 1-specificity) were 0.593 (0.763, 0.170), 0.630 (0.748, 0.118) and 0.821 (0.858, 0.036) at 10, 11, and 14 years respectively. The corresponding threshold distances were 1421 m, 1627 m and 3046 m. Sensitivity analysis restricting the sample to those participating at all three time points did not substantially alter the conclusions drawn (data not shown).

## 4 Discussion

This study shows that young people living closer to school are more likely to walk to school than those living further away (Panter et al., 2008; Pont et al., 2009). The novel contribution of this work is the identification of the threshold distances that children walk to school, and that this increases as young people age; the criterion distances were 1421 m at 10 years, 1627 m at 11 years and 3046 m at 14 years.

Although the distance from home to school has previously been identified as a key predictor of active commuting to school (Davison et al., 2008), little evidence is available on the distance are young people walk to school. To our knowledge, only one study determined a threshold distance among children (D’Haese et al., 2011) and two studies among adolescents (Nelson et al., 2008; Van Dyck et al., 2010); the threshold distances varied between 1.5 km in Belgium children and 2 km/2.4 km in Belgium/Irish adolescents. These studies calculated the criterion distance using descriptive data, based on the distance over which 80% of the walkers lived.

Despite ROC curve analyses are being widely used in biomedical research, their application to identify the distance that best discriminates walkers and passive commuters is novel. The high values obtained for the area under the curve in the present study (all >0.872), showed the accuracy and appropriateness of the ROC curves to discriminate walkers from passive travellers regarding the distance from home to school. This approach provides a further step for calculating the actual walkable distance, since the previously mentioned studies (D’Haese et al., 2011; Nelson et al., 2008; Van Dyck et al., 2010) only used descriptive data.

The distance best discriminating walkers from passive commuters was identified as 1.4 km and 1.6 km when participants were 10 and 11 years old respectively, and increased up to 3 km when participants were 14 years old. These results match with previous cross-sectional evidence in Belgium children and adolescents (D’Haese et al., 2011; Van Dyck et al., 2010). It likely reflects higher independent mobility in adolescents compared to children and the fact that secondary school students commonly live further away from school than primary school-aged children. This is confirmed here by a median distance to primary school of 1370 m (interquartile range: 702–2855 m) compared to 3901 m (1477–7776 m) to secondary school. A “walkable” distance is commonly used in built environment and active living research to define “neighbourhood” buffers (e.g., 800 m, 1 km, 1.6 km). Based on the research shown here, it is likely that these buffer sizes may vary across age-groups. The appropriate definition of “neighbourhood” for population subgroups therefore requires further investigation.

Distance to school has been positively associated with both 1-year maintenance and take up of active commuting within this sample (Panter et al., 2013). Consequently, the reported change of the walkable distance with age in the current study should be taken into account when planning interventions to increase the rates of active commuting to school. These interventions should consider two approaches: (a) increase the rate of walkers within the currently identified walkable distance (i.e., those living closer to 1.5 km among children aged 10 years) or (b) increase the length of the threshold distance (i.e., targeting active travel interventions at 10-year old children living between 1.5 and 2.0 km). The current findings also contribute to progressing research translation from public health through to urban design and planning (Koohsari et al., 2013). Identifying threshold distances is important to help urban designers/planners develop neighbourhoods that support active commuting to school (i.e., to locate the schools within walkable distances from residential areas) and feed into policy decisions around school commuting (i.e., set the cut-offs points for school bus provision).

The main strength of the work presented here is the longitudinal data from childhood to adolescence. Limitations include the sample attrition at 1- and 3-year follow-up and the unknown validity of the measure of active travel. We recognise the importance of perceived distance, which is likely to differ from objectively-measured distance and have a distinct influence. Future work exploring the unique influence of perceived and actual distance is warranted. Lastly, the results may only be generalisable to similar environmental settings.

In conclusion, our results show that the threshold distance that young people walk changes as they get older; from 1.4 km at 10 years, 1.6 km at 11 years to 3 km at 14 years. Future interventions for increasing active modes of commuting to school should take into account these threshold distances.

## Figures and Tables

**Table 1:** Descriptive characteristics of the SPEEDY sample at baseline (10 years), 1-year follow-up (11 years), and 4-year follow up (14 years).

	Baseline (*n*=2053)	1-year follow-up (*n*=911)	4-year follow-up (*n*=475)
	*N* (%)	*N* (%)	*N* (%)
Child/Adolescent characteristics			
Age (years)	10.3 (0.3)	10.6 (1.2)	14.3 (0.3)
BMI (kg/m^2^)	18.22 (3.18)	–	20.9 (4.0)
Sex			
Male	919 (44.8)	370 (40.6)	215 (45.3)
Female	1134 (55.2)	531 (58.3)	260 (54.7)
Mode of commuting to school			
Walk	814 (39.6)	420 (46.1)	164 (34.5)
Bicycle	189 (9.2)	72 (7.9)	21 (4.4)
Car	923 (45.0)	357 (39.2)	120 (25.3)
Bus or Train	127 (6.2)	62 (3.0)	170 (35.8)
Parental characteristics			
Age left full-time education			
<16 years	901 (48.0)	–	–
16–18 years	603 (32.1)	–	–
Over 18 years	374 (19.9)	–	–
Household characteristics			
Distance to school (m)^a^	1370 (702, 2855)	1321 (660, 2788)	3901 (1477, 7776)
Urban/Rural status			
Urban	1366 (67.9)	–	297 (62.7)
Rural	646 (32.1)	–	177 (37.3)

a Expressed as median (25th, 75th) percentile. At 1-year follow-up the distance to school from baseline was used after eliminating those 42 participants who moved from baseline.

**Table 2:** Odds ratio for walking to school (vs. passive) according to distance from home to school (in quintiles) at 10 years (*n*=1666), at 11 years (*n*=746) and at 14 years (*n*=356).

Distance (metres)	*N*	OR	95% CI	*P*
**10 years old**⁎				
3721.0–40,938.4	364	1	Reference	
1784.5–3720.9	334	5.22	2.72–10.01	<0.001
1059.1–1784.4	313	20.45	10.85–38.54	<0.001
592.9–1059.0	316	44.61	23.64–84.19	<0.001
≤592.8	339	223.03	112.66–441.51	<0.001
**11 years old**⁎⁎				
3621.9–33,493.0	162	1	Reference	
1741.5–3621.8	143	16.86	4.97–57.13	<0.001
1033.3 –1741.4	139	63.10	18.57–214.37	<0.001
550.0 –1033.2	143	126.14	37.01–429.82	<0.001
≤549.9	159	582.22	156.39–2167.52	<0.001
**14 years old**⁎⁎⁎				
8406.1–45,767.8	76	1	Reference	
5298.6–8406.0†	73	–	–	0.997
2500.8–5298.5	67	14.05	1.71–115.21	<0.014
1308.1–2500.7	69	155.64	19.12–1266.58	<0.001
≤1308.0	71	265.88	31.12–2271.42	<0.001

⁎ Analysis was adjusted for sex (*p*=0.008), BMI (*p*=0.024), parent׳s educational level (*p*=0.125) and urban/rural status (*p*<0.001).

⁎⁎ Analysis was adjusted for sex (*p*=0.907), and parent׳s educational level (*p*=0.807) and urban/rural status (*p*=0.003) at baseline.

⁎⁎⁎ Analysis was adjusted for sex (*p*=0.451), BMI (*p*=0.784), urban/rural status (*p*=0.003) and parent׳s educational level at baseline (*p*=0.648).

† The model did not converge due to low number of walkers.

## References

[bib1] Bibby P., Shepherd J. (2004). Developing a New Classification of Urban and Rural Areas for Policy Purposes: The Methods.

[bib2] Black C., Collins A., Snell M. (2001). Encouraging walking: the case of journey-to-school trips in compact urban areas. Urban Stud..

[bib3] Buliung R.N., Mitra R., Faulkner G. (2009). Active school transportation in the Greater Toronto area, Canada: an exploration of trends in space and time (1986–2006). Prev. Med..

[bib4] Corder K., Sharp S.J., Atkin A.J., Griffin S.J., Jones A.P., Ekelund U., VanSluijs E.M. (2014). Change in objectively measured physical activity during the transition to adolescence. Br J Sports Med..

[bib5] D’Haese S., De Meester F., De Bourdeaudhuij I., Deforche B., Cardon G. (2011). Criterion distances and environmental correlates of active commuting to school in children. Int. J. Behav. Nutr. Phys. Act..

[bib6] Davison K.K., Werder J.L., Lawson C.T. (2008). Children׳s active commuting to school: current knowledge and future directions. Prev. Chronic Dis..

[bib7] Hume C., Timperio A., Salmon J., Carver A., Giles-Corti B., Crawford D. (2009). Walking and cycling to school: predictors of increases among children and adolescents. Am. J. Prev. Med..

[bib8] Komori O., Eguchi S. (2010). A boosting method for maximizing the partial area under the ROC curve. BMC Bioinform..

[bib9] Koohsari M.J., Badland H., Giles-Corti B. (2013). (Re)Designing the built environment to support physical activity: bringing public health back into urban design and planning. Cities.

[bib10] Larouche R., Saunders T.J., Faulkner G., Colley R., Tremblay M. (2014). Associations between active school transport and physical activity, body composition, and cardiovascular fitness: a systematic review of 68 studies. J. Phys. Act. Health.

[bib11] McDonald N.C. (2007). Active transportation to school: trends among U.S. schoolchildren, 1969–2001. Am. J. Prev. Med..

[bib12] Nelson N.M., Foley E., O’Gorman D.J., Moyna N.M., Woods C.B. (2008). Active commuting to school: how far is too far?. Int. J. Behav. Nutr. Phys. Act..

[bib13] Ordenance, Survey, 2006. OS MasterMap Address Layer.

[bib14] Panter J., Corder K., Griffin S.J., Jones A.P., van Sluijs E.M. (2013). Individual, socio-cultural and environmental predictors of uptake and maintenance of active commuting in children: longitudinal results from the SPEEDY study. Int. J. Behav. Nutr. Phys. Act..

[bib15] Panter J.R., Jones A.P., van Sluijs E.M. (2008). Environmental determinants of active travel in youth: a review and framework for future research. Int. J. Behav. Nutr. Phys. Act..

[bib16] Pont K., Ziviani J., Wadley D., Bennett S., Abbott R. (2009). Environmental correlates of children׳s active transportation: a systematic literature review. Health Place.

[bib17] Schisterman E.F., Perkins N.J., Liu A., Bondell H. (2005). Optimal cut-point and its corresponding Youden Index to discriminate individuals using pooled blood samples. Epidemiology.

[bib18] Tudor-Locke C., Ainsworth B.E., Popkin B.M. (2001). Active commuting to school—an overlooked source of childrens׳ physical activity?. Sports Med..

[bib19] van der Ploeg H.P., Merom D., Corpuz G., Bauman A.E. (2008). Trends in Australian children traveling to school 1971–2003: burning petrol or carbohydrates?. Prev. Med..

[bib20] Van Dyck D., De Bourdeaudhuij I., Cardon G., Deforche B. (2010). Criterion distances and correlates of active transportation to school in Belgian older adolescents. Int. J. Behav. Nutr. Phys. Act..

[bib21] Van Sluijs E.M., Skidmore P.M., Mwanza K., Jones A.P., Callaghan A.M., Ekelund U., Harrison F., Harvey I., Panter J., Wareham N.J., Cassidy A., Griffin S.J. (2008). Physical activity and dietary behaviour in a population-based sample of British 10-year old children: the SPEEDY study (Sport, physical activity and eating behaviour: environmental determinants in young people). BMC Public Health.

